# Is There Any Association Between Psoriasis and Hashimoto’s Thyroiditis?

**DOI:** 10.7759/cureus.4269

**Published:** 2019-03-19

**Authors:** Haider A Alidrisi, Khalil Al Hamdi, Abbas A Mansour

**Affiliations:** 1 Diabetes and Endocrinology, University of Basrah College of Medicine, Basrah, IRQ; 2 Dermatology, University of Basrah College of Medicine, Basrah, IRQ

**Keywords:** psoriasis, psoriasis, hashimoto’s thyroiditis, antithyroid peroxidase antibodies, thyroid ultrasound

## Abstract

Background

The association between psoriasis and Hashimoto’s thyroiditis has been evaluated in many retrospectives and prospective studies with varying numbers of patients and study designs. A positive association had been found certain studies, while no clear association in others.

Objective

The objective of this study was to evaluate the prevalence of Hashimoto’s thyroiditis in patients with psoriasis in comparison with healthy matched control from the same geographical region.

Methods

A case-control study was conducted from October 2017 to October 2018 in Faiha Specialized Diabetes, Endocrine, and Metabolism Center (FDEMC). Fifty-six psoriatic patients were compared with 54 healthy, gender, age and body mass index-matched controls. All participants had thyroid evaluation in the form of measurement of thyroid-stimulating hormone (TSH), free thyroxine (FT4), antithyroid peroxidase antibody (TPO Ab), and antithyroglobulin antibody (Tg Ab). Thyroid ultrasound examination was performed looking for volume, hypo-echogenicity, pseudo-nodularity, and increased vascularity. Assessment of psoriasis severity was conducted using the Psoriasis Area and Severity Index (PASI) score.

Results

Significantly higher prevalence of TPO Ab, Tg Ab, hypo-echogenicity, pseudo-nodularity, and increased vascularity was found in patients with psoriasis. The prevalence in psoriasis versus control was for TPO Ab (25.0% vs 9.3%, *p* = 0.02), Tg Ab (30.4% vs 11.1%, *p* = 0.01), hypo-echogenicity (30.4% vs 9.3%, *p* = 0.02), pseudo-nodularity (16.1% vs 0%, *p* = 0.002), and increased vascularity (35.7% vs 5.6%, *p* = 0.001). Patients with psoriasis with age of onset at diagnosis ≥40 years old and obesity were significantly more likely to have positive TPO Ab with a prevalence of (42.1% and 40.7%, respectively). There were no significant differences in the prevalence of hypothyroidism and subclinical hypothyroidism between psoriasis and control. In patients with psoriasis, psoriasis types, severity, duration, age, gender, smoking status, type 2 diabetes, and personal and family history of autoimmune diseases did not correlate with thyroid autoimmunity.

Conclusions

This study demonstrates a clear association between psoriasis and Hashimoto’s thyroiditis in the form of a significantly higher prevalence of TPO Ab, Tg Ab, hypo-echogenicity, pseudo-nodularity, and increased vascularity. Hence, thyroid evaluation by anti-thyroid antibodies, particularly TPO Ab, and ultrasound should be included in the care of psoriasis patients.

## Introduction

Psoriasis is a complex autoimmune-mediated inflammatory disease that affects individuals with genetic susceptibility and influenced by environmental triggers [1]. Many studies showed that autoimmune diseases are more prevalent in patients with psoriasis than in the general population. The common signaling pathway may be implicated in the pathophysiology of these autoimmune diseases [2].

The association of Hashimoto’s thyroiditis and psoriasis has been evaluated in many retrospectives and prospective studies with varying numbers of patients and study designs. An increasing prevalence of thyroid antibodies, hypothyroidism, and ultrasound finding of Hashimoto’s thyroiditis have been found in patients with psoriasis and psoriatic arthritis in certain studies, while no clear association had been found in others [3].

At the cellular level, many of the pathophysiological mechanisms are shared between psoriasis and Hashimoto’s thyroiditis. It has been found that INF-gamma and Th1 cytokines/chemokines, importantly C-X-C motif chemokine 10 (CXCL10), IL-23, and Th17 are involved in the pathogenesis of psoriasis and psoriatic arthritis [4-5]. In the same time, autoimmune thyroid diseases including Hashimoto’s thyroiditis are Th1 immune-mediated disorder, in which Th1 cells, IFN-gamma with its dependent chemokines including CXCL10 play an important role [6]. Furthermore, a significantly higher level of CXCL10 has been found in psoriatic arthritis with autoimmune thyroid diseases than psoriatic arthritis alone [7]. Another two cytokines, which are IL-23 and Th17, have been found with increased levels both in psoriasis and Hashimoto’s thyroiditis, suggesting the possibility for the involvement of these two cytokines in immunopathogenesis for both diseases [8]. Another possible pathophysiological linkage is that psoriatic skin lesion has abnormal thyroid hormone and retinoid signaling [9].

Besides all these pathophysiological factors, thyroid hormones take part in the pathogenesis of psoriasis. Thyroid hormones receptors are expressed in the skin and may trigger increased epidermal growth factor, which in turn will promote keratinocytes proliferation and differentiation, a manifestation relevant to psoriasis. Besides, the severity of psoriasis may be correlated with thyroid hormones levels. For this reason, thyroid hormones may be implicated in the pathogenesis and severity of psoriasis [10-11].

In another way, patients with thyroid diseases including Hashimoto’s thyroiditis have a wide range of cutaneous manifestations which put thyroid diseases in the field of interest for both endocrinologists and dermatologists. Many cutaneous manifestations are independent of thyroid function and mainly related to autoimmune thyroid diseases like Sjögren’s syndrome, systemic lupus erythematosus, systemic sclerosis, vitiligo, alopecia areata, diffuse alopecia, chronic urticaria, idiopathic hirsutism, and pre-menstrual acne [12]. While other manifestations may be related directly to thyroid hormone deficiency including cold skin, skin dryness, puffiness of eyes, palmoplantar keratoderma, and purpura. The latter usually disappear, improve, or sometimes persist with thyroxine replacement [13].

The aim of this study is to evaluate the prevalence of Hashimoto’s thyroiditis in patients with psoriasis in comparison with healthy gender, age, and body mass index (BMI)-matched control from the same geographical region.

## Materials and methods

**Figure 1:**
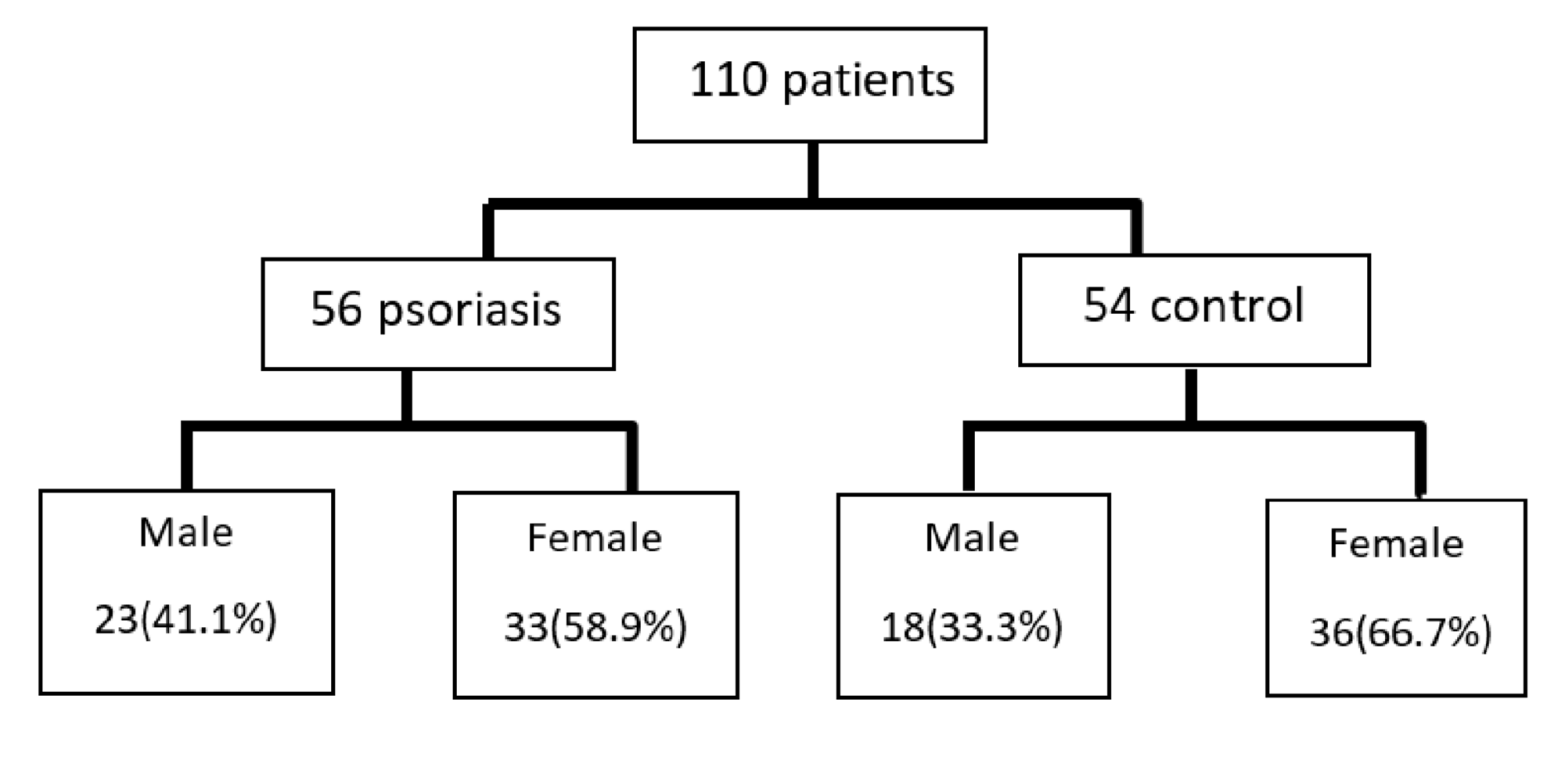
Scheme of the patients enrolled in the study

A case-control study was conducted in Faiha Specialized Diabetes, Endocrine and Metabolism Center (FDEMC), which is a tertiary referring center for diabetes, endocrine, and metabolism in Basrah (Southern Iraq), during the period from October 2017 to October 2018. The scheme of the patients enrolled in the study is shown in Figure 1.

We had 56 patients with psoriasis were included, the youngest being 12 years old and the eldest being 71 years old. Those patients were compared with 54 healthy, gender, age, and BMI-matched controls. All participants enrolled were from Basrah and given a written informed consent before proceeded in full study evaluation.

Clinical data 

All of the patients with psoriasis were undergone evaluation by a dermatologist before referral for collection of disease-related clinical data in the form of psoriasis duration, psoriasis types including (plaque, erythrodermic, inverse, guttate, pustular, nail, and psoriatic arthritis). The severity of psoriasis was assessed by calculating two parameters [14]:

1. The percentages of body surface areas (BSA) involvement in the form of the head, arms, trunk, legs, and total BSA.

2. Psoriasis Area and Severity Index score (PASI score).

For all participants, history and examination were taken looking for exclusion criteria and clinical data collection in the form of type 2 diabetes, smoking status, personal and family history of autoimmune diseases. Weight and height measurements were performed with bare feet and light clothes, and BMI was calculated by dividing body weight in kilogram (kg) by height (square meter; m^2^)

Exclusion criteria

1. Currently hospitalized patients

2. History of liver and/or renal diseases

3. Pregnancy within one year

4. Any history of autoimmune thyroid diseases

5. Patients on drugs that interfere with thyroid hormones levels including, dopamine, levodopa, bromocriptine, glucocorticoids (>0.5 mg/day dexamethasone, 100 mg/day hydrocortisone), octreotide, amphetamines, metoclopramide, amiodarone, iodinated contrast media, intravenous furosemide >80 mg/day, non-steroidal anti-inflammatory drugs, Intravenous heparin, phenytoin, carbamazepine, lithium, and INF-alpha [15].

6. Exposure to medical and/or accidental irradiation.

Hormonal and antibodies assessment

From each participant, 10 ml of blood was taken and put in clot activator tube, centrifuged immediately, serum was separated, and then frozen at -20 °C to be stored for analysis. Thyroid stimulating hormone (TSH), free thyroxine (FT4), antithyroid peroxidase antibody (TPO Ab), and antithyroglobulin antibody (Tg Ab) were analyzed by Electro-Chemi Luminescence (ECL) assay (Cobas e 411 analyzer - Roche, Germany). Normal value for TSH (0.27-4.2 µIU/ml) with a measuring range of (0.005-100.0 μIU/ml), FT4 (0.93-1.7 ng/dL) with a measuring range of (0.023-7.77 ng/dl), TPO Ab (<34 IU/mL) with a measuring range of (5-600 IU/mL), and Tg Ab (<115 IU/mL) with a measuring range (10-4000 IU/mL). Hashimoto’s thyroiditis was diagnosed by the presence of a high level of these antibodies. Hypothyroidism was diagnosed when TSH level above normal and FT4 level less than normal. Subclinical hypothyroidism was diagnosed when the TSH level was above normal with normal FT4 level [16].

Thyroid ultrasound examination

All participants have undergone thyroid ultrasound examination by one examiner before gaining the laboratory results using (A high-resolution fully equipped ultrasound and Doppler ultrasound device (GE Logiq P5) with a 10 MHz broadband linear array transducer was used for the examinations.

Thyroid ultrasound examinations were done in grayscale and color Doppler images looking for findings of Hashimoto’s thyroiditis [17-18]:

1. Measurement of thyroid gland dimension in three axes. Thyroid lobes volumes were calculated by (anteroposterior x mediolateral x craniocaudal diameters x 0.479).

2. In grayscale images, assessment for the presence of pseudo-nodularity and echogenicity was done. Pseudo-nodularity is characterized by the presence of multiple multifocal hypoechoic lesions within the thyroid parenchyma with varying shapes and without a clear margin to the parenchyma. The echogenic patterns were graded in comparison to the surrounding muscles as normal, grade I with a slight reduction in echogenicity, grade III with a marked reduction in echogenicity to less than that of surrounding muscles, and grade II between grade I and II).

3. Color Doppler scale appearance in the form of (grade 0 with parenchymal flow minimal or absent, grade one with a mild increase in parenchymal flow, grade two with a clear increase in parenchymal flow, and grade three with a marked increase in parenchymal flow).

Statistical analysis

The Statistical Package for the Social Sciences (SPSS) Version 23.0. was used for analysis. Qualitative variables were summarized as numbers (N) and frequencies (%), while quantitative variables were summarized as mean and standard deviation (M ± SD). Independent Student’s t-test was used for correlating between qualitative and quantitative variables. Chi-squared test was used for in between qualitative variables correlations, odds ratio (OR) and 95% confidence interval (95%CI) calculations. For all the tests performed, results were considered statistically significant if (*P*-value < 0.05).

## Results

Table 1 summarizes the general characteristics of the patients with psoriasis and control and shows comparable frequencies for these general characteristics between the two groups. In patients with psoriasis, 33 (58.9%) were females with a female to male ratio of (1.43:1). The mean BMI was 29.84 ± 6.60 kg/m^2^, and 27 were obese with a prevalence of obesity of (48.2%) in patients with psoriasis. The most common type of psoriasis in the study was plaque psoriasis in 46 patients (82.2%), and this was followed by guttate in seven patients (12.5%) and erythrodermic only in three patients (5.3%). From patients with plaque psoriasis, eight had psoriatic arthritis and five had nail psoriasis, which both represent a prevalence of (14.2%) and (8.9%), respectively, from the total psoriasis group. A positive family history of autoimmune thyroid diseases was present in three (5.4%) and four (7.4%) of psoriasis and control, respectively. Personal history of autoimmune diseases was present only in psoriasis and in three patients (5.4%) which included rheumatoid arthritis, type 1 diabetes, celiac disease, and vitiligo.

**Table 1:** General characteristics of the patients with psoriasis and control *Diagnosed in patients with plaque psoriasis and four patients have both arthritis and nail psoriasis BMI, body mass index; T2DM, type 2 diabetes; RA, rheumatoid arthritis; T1DM, type 1 diabetes; HT, Hashimoto’s thyroiditis; AITD, autoimmune thyroid disease

	Psoriasis = 56	Control = 54	P value
	M±SD or N (%)	M±SD or N (%)	
Age (years)	43.05±16.72	41.28±14.78	0.58
Age ≥45 years	26 (46.4)	22 (40.7)	0.54
Age at onset (years)	31.71±18.23		
Age at onset ≥40 years old	19 (33.9)		
Duration of psoriasis (years)	11.88±10.66		
Plaque	46 (82.2)		
Guttate	7 (12.5)		
Erythrodermic	3 (5.3)		
Psoriatic arthritis*	8 (14.3)		
Nail psoriasis*	5 (8.9)		
Male	23 (41.1)	18 (33.3)	0.40
Female	33 (58.9)	36 (66.7)	
BMI (kg/m^2^)	29.84±6.60	28.84±7.04	0.44
BMI ≥ 30 kg/m^2^	27 (48.2)	23 (42.6)	0.55
Current smoker	4 (7.1)	4 (7.4)	0.91
Former-smoker	4 (7.1)	5 (9.3)	
Never-smoker	48 (85.7)	45 (83.3)	
T2DM	15 (26.8)	13 (24.1)	0.74
Personal history of autoimmune diseases	3 (5.4)	0 (0)	0.08
RA	1 (1.8)	0 (0)	
T1DM and celiac disease	1 (1.8)	0 (0)	
Vitiligo	1 (1.8)	0 (0)	
Family history of autoimmune diseases	4 (7.1)	4 (7.4)	0.95
Graves’ disease	1 (1.8)	0 (0)	
HT	2 (3.6)	4 (7.4)	0.44
Vitiligo	1 (1.8)	0 (0)	
Family history AITD	3 (5.4)	4 (7.4)	0.66

Thyroid function did not vary significantly between psoriasis and control, as shown in Table 2. Hypothyroidism was diagnosed in three (5.4%) and subclinical hypothyroidism was diagnosed in five (8.9%) patients with psoriasis as compared to one (1.9%) with hypothyroidism and four (7.4%) with subclinical hypothyroidism in control, (*P* = 0.58). The mean TSH was higher and mean FT4 was lower in patients with psoriasis, but with no statistical significance, (*P *= 0.07). On the other hand, thyroid antibodies correlated significantly with psoriasis, including TPO Ab positive in 14 (25%) and Tg Ab positive 17 (30.4%), with only five (9.3%) TPO Ab positive, and six (11.1%) Tg Ab positive in control (OR 3.2 [1.08-9.82], *P *= 0.02 and 3.4 (1.25-9.69), *P *= 0.01) for TPO Ab and Tg Ab, respectively. Also, both antibodies were positive in 12 (21.4%) of patients with psoriasis as compared to four (7.4%) in control, (OR 3.4 [1.02-11.34], *P *= 0.03). While the mean level for TPO Ab was significantly higher in psoriasis (*P *= 0.03), the level of Tg Ab did not differ significantly in between groups.

**Table 2:** Thyroid functionality and autoimmunity between patients with psoriasis and control SCH, subclinical hypothyroidism; TSH, thyroid-stimulating hormone; FT4, free thyroxine; TPO Ab, antithyroid peroxidase antibody; Tg Ab, antithyroglobulin antibody

	Psoriasis N (%) or M ± SD	Control N (%) or M ± SD		P value
Euthyroid	48 (85.7)	49 (90.7)		
SCH	5 (8.9)	4 (7.4)		0.58
Hypothyroid	3 (5.4)	1 (1.9)		
TSH (µIU/mL)	4.67 ± 12.19	1.69 ± 1.30		0.07
FT4 (ng/dL)	1.13±0.26	1.23 ± 0.33		0.07
			OR (95%CI)	
TPO Ab +ve	14 (25.0)	5 (9.3)	3.2 (1.08-9.82)	0.02
TPO Ab (IU/mL)	64.12 ± 139.80	23.13 ± 24.86		0.03
Tg Ab +ve	17 (30.4)	6 (11.1)	3.4 (1.25-9.69)	0.01
Tg Ab (IU/mL)	254.65 ± 668.61	168.15 ± 709.56		0.51
TPO+Tg Ab +ve	12 (21.4)	4 (7.4)	3.4 (1.02-11.34)	0.03

Thyroid ultrasound findings were shown to have significant correlations with psoriasis, as shown in Table 3. Reduced parenchymal echogenicity in all grades was significantly more found in psoriasis as compared to control. In patients with psoriasis, 17 (30.4%) had reduced echogenicity, grade-I 10 (17.9%), grade II three (5.4%), and grade-III four (7.1%) in comparison to only five (9.3%) in control had reduced echogenicity and only in grade I, (*P* = 0.02). While pseudo-nodularity is found in nine (16.1%) of patients with psoriasis, none of the control group had it on ultrasound, (*P *= 0.002). Increased vascularity on color Doppler ultrasound also had a similar correlation with psoriasis. Increased vascularity was found in 20 (35.7%) patients with psoriasis, including 12 grade one patients (21.4%), five in grade two (8.9%), and grade three (5.4%). However, only three (5.6%) had increased vascularity and only in grade one in control (*P* = 0.001). The calculated thyroid lobes volume and total thyroid volume were higher but non-significantly in psoriasis than control.

**Table 3:** Correlation of thyroid ultrasound findings of patients with psoriasis and control

	Psoriasis = 56	Control = 54	
	M ± SD or N (%)	M ± SD or N (%)	P value
Thyroid volume (ml)			
Right Lobe	6.50±7.96	4.28±1.41	0.04
Left Lobe	5.58±5.73	4.22±1.55	0.09
Total	12.08±13.60	8.51±2.62	0.06
Echogenicity			
Normal	39 (69.6)	49 (90.7)	0.02
Grade-I	10 (17.9)	5 (9.3)	
Grade-II	3 (5.4)	0 (0)	
Grade-III	4 (7.1)	0 (0)	
Pseudo-nodularity			
Normal	47 (83.9)	54 (100)	0.002
Pseudo-nodular	9 (16.1)	0 (0)	
Vascularity			
Grade-0	36 (64.3)	51 (94.4)	0.001
Grade-1	12 (21.4)	3 (5.6)	
Grade-2	5 (8.9)	0 (0)	
Grade-3	3 (5.4)	0 (0)	

**Figure 2:**
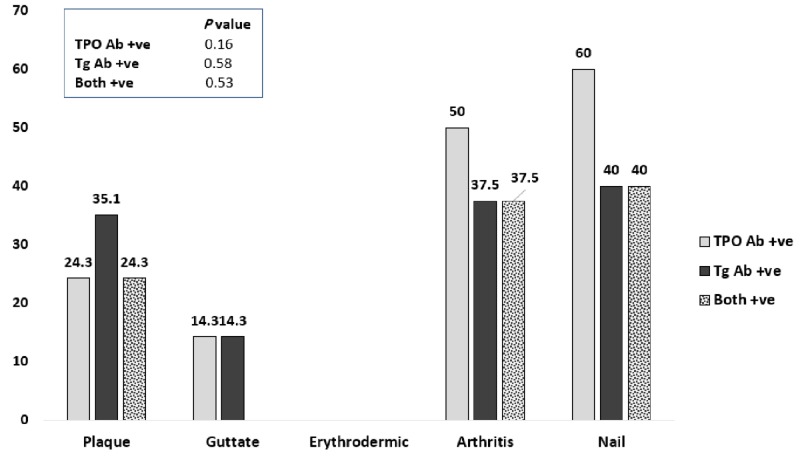
Percentages of thyroid antibodies within different types in patients with psoriasis

Patients were more likely to have positive thyroid antibodies in psoriatic arthritis and nail psoriasis, as shown in Figure 2. From eight psoriatic arthritis patients, four (50%) had positive TPO Ab and three (37.5%) had Tg Ab positive. From five patients with nail psoriasis and from whom four (80%) had associated psoriatic arthritis, TPO Ab and Tg Ab positive in three (60%) and two (40%), respectively. These frequencies were relatively higher in a ratio of 2:1 as compared to the overall psoriasis group.

While TPO Ab and Tg Ab were correlated significantly with psoriasis, within psoriasis both obesity and age at onset of psoriasis (≥40 years) old had a significantly higher prevalence of TPO Ab (*P* = 0.009 and 0.03, respectively), as shown in Table 4. Obese patients with psoriasis had positive TPO Ab in 11 (40.7%), and patients with psoriasis with age at onset of (≥40 years) old had positive TPO Ab in eight (42.1%). Other confounders, in the form of age, gender, type two diabetes, smoking status, and personal and family history of autoimmune diseases did not correlate significantly with TPO Ab and Tg Ab in patients with psoriasis.

**Table 4:** Correlations of thyroid antibodies prevalence with confounders within patients with psoriasis *Percentages are within cofounders. TPO Ab, antithyroid peroxidase antibody; Tg Ab, antithyroglobulin antibody; BMI, body mass index; T2DM, type two diabetes mellitus

	TPO Ab +ve N (%)	Tg Ab +ve N (%)
Age		
≥ 45 years	9 (34.6)	10 (38.5)
<45 years	5 (16.7)	7 (23.3)
P value	0.12	0.29
Age at onset of diagnosis		
<40 years	6 (16.2)	8 (21.6)
≥ 40 years	8 (42.1)	9 (47.4)
P value	0.03	0.06
Male	5 (21.7)	6 (26.1)
Female	9 (27.3)	11 (33.3)
P value	0.63	0.56
BMI ≥ 30 kg/m^2^	11 (40.7)	10 (37.0)
BMI < 30 kg/m^2^	3 (10.3)	7 (24.1)
P value	0.009	0.29
T2DM	5 (33.3)	5 (33.3)
Non-T2DM	9 (22.0)	12 (29.3)
P value	0.38	0.77
Current smoker	1 (25)	2 (50)
Former-smoker	1 (25)	1 (25)
Never-smoker	12 (25)	14 (29.2)
P value	0.99	0.66
Personal history of autoimmune diseases		
Yes	1 (33.3)	1 (33.3)
No	13 (24.5)	16 (30.2)
P value	0.73	0.90
Family history of autoimmune diseases		
Yes	1 (25.0)	1 (25.0)
No	13 (25.0)	16 (30.8)
P value	0.73	0.64

The severity features of psoriasis in the form of PASI score, BSA, and duration of psoriasis had no significant correlation with TPO Ab, Tg Ab positivity nor with thyroid ultrasound findings of reduced echogenicity, pseudo-nodularity, and increased vascularity (Table 5).

**Table 5:** Correlation of PASI score, BSA and duration with thyroid autoimmunity and ultrasound thyroid findings PASI, psoriasis area and severity index; BSA, body surface area; TPO Ab, antithyroid peroxidase antibody; Tg Ab, antithyroglobulin antibody

	PASI score M±SD	BSA (%) M±SD	Duration (years) M±SD
TPO Ab			
TPO Ab +ve	12.59±15.41	21.81±27.20	10.78±9.03
TPO Ab -ve	12.44±10.24	23.14±25.55	12.26±11.24
P value	0.96	0.86	0.65
Tg Ab			
Tg Ab +ve	12.83±13.88	21.78±24.53	10.64±8.82
Tg Ab -ve	12.33±10.64	23.25±26.53	12.44±11.45
P value	0.88	0.84	0.56
Echogenicity			
Normal	12.05±9.84	22.19±23.20	12.56±10.90
Reduced	13.46±15.17	24.22±31.53	10.37±10.26
P value	0.68	0.78	0.48
Pseudo-nodularity			
Normal	13.74±12.04	25.06±26.87	12.32±11.20
Pseudo-nodular	5.90±5.59	11.03±14.63	9.66±7.41
P value	0.06	0.13	0.49
Vascularity			
Minimal or absent	13.17±10.94	25.30±26.77	11.75±10.98
Increase	11.24±12.87	18.32±23.71	12.12±10.34
P value	0.55	0.33	0.90

## Discussion

Many studies have evaluated the association between psoriasis and/or psoriatic arthritis with Hashimoto’s thyroiditis with mixing results [19-24].

Bianchi et al. have studied the association between thyroid disease and psoriatic arthritis in a retrospective study and found an increased prevalence of TPO Ab in psoriatic arthritis patients in comparison to control. Also, the average thyroid volume measured by ultrasound was higher among psoriatic arthritis patients [24]. In this study, thyroid volume was higher but non-significantly among patient with psoriasis. A similar association also have been observed by Antonelli et al. who found a significantly higher prevalence of TPO Ab and higher frequency of hypo-echogenic thyroid on ultrasound among psoriatic arthritis patients in comparison with age and gender-matched control. Also, they found that women with psoriatic arthritis have a significantly higher frequency of subclinical hypothyroidism with a prevalence of 25%, and those patients with subclinical hypothyroidism have a longer psoriasis disease duration [19]. In this study, the sum prevalence of both subclinical hypothyroidism and hypothyroidism was 14.2% which did not differ significantly from the control. A more recent study with a prospective design was aimed to compare the incidence of new cases of thyroid diseases between patients with psoriatic arthritis and age and gender-matched control after a median follow up of six years. A significant increase in the incidence of subclinical hypothyroidism, TPO and Tg Abs positive, and hypo-echogenicity on thyroid ultrasound was present in patients with psoriatic arthritis, especially among women [20].

In our study, a comparable increased prevalence of TPO and Tg Abs positivity and thyroid ultrasound hypo-echogenicity with the aforementioned three studies. In addition to hypo-echogenicity on thyroid ultrasound, two other ultrasound findings were examined in this study in the form of pseudo-nodularity and vascularity which were significantly associated with psoriasis. The prevalence of both thyroid antibodies was highest in patients with psoriatic arthritis, but without a significant difference in comparison with psoriasis without arthritis. Furthermore, there was no gender preference in these associations and no significant increase in the frequency of neither subclinical hypothyroidism nor hypothyroidism. This may be explained by the difference in the inclusions criteria for these three studies which included patients with psoriatic arthritis only, while this study included patients with psoriasis with only 14.2% having arthritis.

Three other studies have evaluated the association between psoriasis with or without arthritis and Hashimoto’s thyroiditis. Two of these showed no association. The first study was done by Gul et al. who compared patients with psoriasis without arthritis with age and gender-matched control with tinea pedis and found no significant increase in the prevalence of TPO and Tg Abs in patients with psoriasis [21]. The second was a prospective study including 114 patients with psoriasis, (30 of them had arthritis), to be compared with age and BMI-matched controls. A similar finding has been observed with no significant difference in the prevalence of neither TPO nor Tg Abs positivity between cases and control (20.2% vs. 19.6%); as did not the prevalence of subclinical hypothyroidism and hypothyroidism [23]. The non-significance in the association in the latter study may be explained by the relatively high prevalence of these antibodies in the control group as compared to the prevalence in the general populations [25-26]. Both these two studies also showed that psoriasis severity as assessed by PASI score did not affect the prevalence of thyroid autoimmunity. The third study was a retrospective database study including more than 800,000 individuals and showed a significant association between psoriasis and Hashimoto’s thyroiditis independent of age, gender, psoriatic arthritis, and use of systemic antipsoriatic agents [22].

It is well known that Hashimoto’s thyroiditis is a disease of women [26], but in this study and other studies, there was no gender difference in prevalence of thyroid antibodies among psoriasis and/or psoriatic arthritis [19,22]. Psoriasis may abolish the female gender preference for Hashimoto’s thyroiditis.

In this study, there was a matched prevalence of obesity between psoriasis and control. In patients with psoriasis, a higher prevalence of TPO Ab in obese as compared to non-obese, which was significant (four-folds). It has been found that obesity increases the risk of having autoimmune thyroid diseases with an emerging role for leptin in thyroid autoimmunity [27]. At the same time, previous reports showed that the adipokines-derived cytokines including leptin are present in high concentrations in patients with psoriasis [28]. These findings may explain the higher prevalence of TPO Ab in obese patients with psoriasis.

In this study, a significantly higher prevalence of Hashimoto’s thyroiditis in the form of positive TPO Ab was found in late-onset psoriasis (onset ≥ 40 years old) in comparison with early-onset psoriasis. This finding is difficult to be explained by the cross-sectional design of this study due to the lack of data about the exact onset of the development of thyroid autoimmunity. While Hashimoto’s thyroiditis is associated with HLA class II alleles, class I HLA, specifically HLA-C allele, which is strongly associated with early-onset psoriasis, is not implicated in Hashimoto’s susceptibility. On the other hand, late-onset psoriasis has no clear HLA association [29-30]. Finally, the increased prevalence of thyroid antibodies with increasing age may explain this association [25-26]. 

One of the limitations of this study is that the clear temporal association between psoriasis and Hashimoto’s thyroiditis could not be established due to the cross-sectional design of this study. This requires a larger number of patients and longitudinal studies.

## Conclusions

A significant association was present between psoriasis and Hashimoto’s’ thyroiditis in terms of higher prevalence of TPO Ab and Tg Ab in patients with psoriasis and was not affected by psoriasis types, age, gender, smoking status, type 2 diabetes, and personal and family history of autoimmune diseases. Obesity and age at onset of (≥40 years) for psoriasis were associated with higher risk for development of TPO Ab. Also, patients with psoriasis had a significantly higher prevalence of Hashimoto’s thyroiditis ultrasound features in the form of hypo-echogenicity, pseudo-nodularity, and increased vascularity. Both thyroid ultrasound features and antibodies did not correlate with psoriasis disease duration and severity. This association between psoriasis and Hashimoto’s thyroiditis deserves endocrinologists’ and dermatologists’ attention and may have impacts in the fields of patients’ clinical care and medical researches. In the view of that, we recommend inclusions of thyroid antibodies, particularly TPO Ab, and thyroid ultrasound in the evaluation of patients with psoriasis, especially for those with late-onset psoriasis and obesity.
